# National analysis of racial disparities in emergent surgery for colorectal cancer^☆^

**DOI:** 10.1016/j.sopen.2024.01.013

**Published:** 2024-01-24

**Authors:** Ayesha P. Ng, Nam Yong Cho, Shineui Kim, Konmal Ali, Saad Mallick, Hanjoo Lee, Peyman Benharash

**Affiliations:** a Cardiovascular Outcomes Research Laboratories, David Geffen School of Medicine at UCLA, Los Angeles, CA, USA; b Division of Colon and Rectal Surgery, Department of Surgery, Harbor-UCLA Medical Center, Torrance, CA, USA; c Division of Cardiac Surgery, Department of Surgery, David Geffen School of Medicine at UCLA, Los Angeles, CA, USA

**Keywords:** Colorectal cancer, Racial disparities, Emergent surgery, Outcomes, Access to care

## Abstract

**Background:**

Racial disparities in access to preoperative evaluation for colorectal cancer remain unclear. Emergent admission may indicate lack of access to timely care. The present work aimed to evaluate the association of admission type with race among patients undergoing colorectal cancer surgery.

**Methods:**

All adults undergoing resection for colorectal cancer in 2011–2020 National Inpatient Sample were identified. Multivariable regression models were developed to examine the association of admission type with race. Primary outcome was major adverse events (MAE), including mortality and complications. Secondary outcomes included costs and length of stay (LOS). Interaction terms between year, admission type, and race were used to analyze trends.

**Results:**

Of 722,736 patients, 67.6 % had Elective and 32.4 % Emergent admission. Black (AOR 1.38 [95 % CI 1.33–1.44]), Hispanic (1.45 [1.38–1.53]), and Asian/Pacific Islander or Native American (1.25 [1.18–1.32]) race were associated with significantly increased odds of Emergent operation relative to White. Over the study period, non-White patients consistently comprised over 5 % greater proportion of the Emergent cohort compared to Elective. Furthermore, Emergent admission was associated with 3-fold increase in mortality and complications, 5-day increment in LOS, and $10,100 increase in costs. MAE rates among Emergent patients remained greater than Elective with a widening gap over time. Non-White patients experienced significantly increased MAE regardless of admission type.

**Conclusion:**

Non-White race was associated with increased odds of emergent colorectal cancer resection. Given the persistent disparity over the past decade, systematic approaches to alleviate racial inequities in colorectal cancer screening and improve access to timely surgical treatment are warranted.

## Introduction

Although the incidence and attributable mortality for colorectal cancers have generally declined in the United States over the past several decades, notable racial disparities still persist [1,2]. Compared to Whites, Black patients have a 20 % higher incidence of colorectal cancer and are diagnosed at a younger age and more advanced stage, facing significantly higher mortality [[2], [3], [4]]. While differences in disease physiology and presentation may partly underlie such disparities, variations in disease management significantly contribute and are often challenging to assess. Barriers to surgical care may arise with lower-quality provider evaluation, delayed optimization for surgery, and suboptimal perioperative treatment among racial and ethnic minority patients [5,6].

With timely care as a key indicator of surgical quality, prior literature has suggested emergent admission to be a manifestation of delayed treatment [5,7]. In particular, colorectal cancer is highly conducive to elective surgery due to slow disease progression and standardized screening and treatment algorithms [8,9]. Therefore, emergent surgery for colorectal cancer may reflect inadequate screening and preoperative management. A recent statewide study demonstrated that Black race was associated with increased odds of emergent colorectal cancer surgery [10]. Emergent surgery was further linked with incomplete oncologic evaluation such as preoperative carcinoembryonic antigen measurement and cancer staging, increased postoperative complications, and increased surgical margin positivity. However, a contemporary analysis at the national level encompassing variations in oncologic surgical care across the United States remains lacking.

The present study used a nationally representative cohort of patients with colorectal cancer to evaluate the association of race with the need for emergency surgery over a 10-year period. We further characterized the temporal association of non-elective status on clinical and financial outcomes of colorectal operations. We hypothesized that patients of non-White race would have increased odds of emergent colorectal resection and major adverse events, and that such disparities would persist over time.

## Methods

This was a cross-sectional study using the 2011–2020 National Inpatient Sample (NIS). Maintained by the Healthcare Cost and Utilization Project (HCUP), the NIS is the largest publicly available all-payer inpatient database in the United States [11]. Using robust survey weighting algorithms, the NIS provides accurate estimates for approximately 97 % of all US hospitalizations. All adult patients (≥18 years) with a diagnosis of colon or rectal cancer undergoing colectomy (right, transverse, left, sigmoid, total) or rectal resection were identified using relevant *International Classification of Diseases 9th/10th Revision* (ICD-9/10) diagnosis and procedure codes (Supplemental Table 1).

Patient and hospital characteristics including age, sex, race, primary payer, admission type, and hospital region and teaching status were defined using the HCUP Data Dictionary [11]. Race was grouped into White, Black, Hispanic, or Other (including Asian or Pacific Islander and Native American). The NIS-provided binary variable “ELECTIVE” was used to identify whether an operation was elective or non-elective, which includes both emergent and urgent cases [12]. For the purposes of the present study, both emergent and urgent procedures were grouped into the *Emergent* cohort, while elective operations were categorized as *Elective*. Records with missing data for admission type, race, age, sex, payer, or mortality were excluded from analysis.

Comorbidities such as diabetes, hypertension, obesity, congestive heart failure, chronic kidney disease, chronic liver disease, and anemia were identified using ICD-9/10 diagnosis codes (Supplemental Table 1). The Elixhauser Comorbidity Index, a validated composite of 30 comorbidities, was additionally used to quantify the overall burden of chronic conditions [13]. Hospitals were stratified into low-volume (<40 cases), medium-volume (40–130 cases), and high-volume (>130 cases) tertiles based on annual institutional case volume of colectomy and rectal resection. ICD-9/10 codes were used to identify minimally invasive operative approaches (laparoscopic and robotic) and postoperative complications, including respiratory (respiratory failure, prolonged mechanical ventilation, pneumonia), infectious (sepsis, abscess, wound infection), renal (acute kidney injury), thromboembolic (deep vein thrombosis, pulmonary embolism), and cardiac (cardiac arrest, myocardial infarction, Supplemental Table 1). Major adverse events (MAE) were defined as a composite of in-hospital mortality and complications. The Clavien–Dindo classification system was used to classify the severity of postoperative complications as no complication/grade I, grade II, grade III, and grade IV/V, according to the ICD algorithm developed by Lentine et al. [14] Hospitalization costs were calculated from charges using hospital-specific cost-to-charge ratios and were inflation adjusted to the 2020 Patient Health Care Index [15]. The primary outcome of interest was MAE, while costs, length of stay (LOS), and non-home discharge were also examined.

Categorical variables are reported as frequencies (%) and compared using the Pearson's chi-square test. Continuous variables are reported as means with standard deviation (SD) or medians with interquartile range (IQR) and compared using the Mann-Whitney U and Kruskal-Wallis tests. Multivariable mixed regression models were developed to evaluate the association of admission type with risk factors and outcomes of interest. Variable selection was performed by applying the Least Absolute Shrinkage and Selection Operator (LASSO) to enhance model generalizability and minimize overfitting and collinearity between independent variables [16]. Interaction terms between year of admission, admission type, and race were included to analyze risk-adjusted temporal trends. Regression results are reported as adjusted odds ratios (AOR) for dichotomous and beta coefficients (β) for continuous variables with 95 % confidence intervals (95 % CI). Statistical significance was set at α = 0.05. All statistical analyses were performed using Stata 16.1 (StataCorp, College Station, TX). This study was deemed exempt from full review by the Institutional Review Board at the University of California, Los Angeles due to the de-identified nature of the NIS.

## Results

Of an estimated 722,736 patients undergoing resection for colorectal cancer, 538,879 (74.6 %) were White, 81,221 (11.2 %) Black, 57,211 (7.9 %) Hispanic, and 45,425 (6.3 %) were categorized as Other race. Compared to Whites, non-White patients were younger (Black: 64 vs Hispanic: 64 vs Other: 65 vs White 70 years) and more commonly had Medicaid (13.0 vs 15.3 vs 13.5 vs 4.7 %) or were uninsured (4.0 vs 5.4 vs 3.6 vs 1.8 %, Table 1). Black patients less frequently received minimally invasive operations (37.1 vs 40.4 %), while Hispanic and Other race patients were less frequently treated at high-volume centers (24.6 vs 31.1 vs 33.4 %) relative to White patients. In addition, Black and Hispanic patients were more common in the South and at metropolitan teaching hospitals (Table 1).

**Table 1:** Patient, operative and hospital characteristics stratified by race and ethnicity. IQR: interquartile range. SD: standard deviation.

Parameter	White (*n* = 538,879)	Black (*n* = 81,221)	Hispanic (*n* = 57,211)	Other (*n* = 45,425)	*p*-Value
Age (years, median, IQR)	70 [60–79]	64 [55–73]	64 [54–74]	65 [55–75]	<0.001
Female sex (%)	49.8	51.8	46.4	47.8	<0.001
Payer status (%)					<0.001
Private	30.4	31.2	30.9	35.8	
Medicare	61.4	48.8	45.1	44.2	
Medicaid	4.7	13.0	15.3	13.5	
Uninsured	1.8	4.0	5.4	3.6	
Other	1.7	3.1	3.3	2.8	
Comorbidities (%)					
Elixhauser Comorbidity Index (mean ± SD)	3.6 ± 1.8	3.8 ± 1.7	3.5 ± 1.7	3.3 ± 1.7	<0.001
Diabetes	22.2	29.1	30.8	26.3	<0.001
Hypertension	58.1	68.3	55.9	53.9	<0.001
Obesity	14.4	17.4	15.8	9.2	<0.001
Chronic kidney disease	0.6	2.4	1.7	1.1	<0.001
Chronic liver disease	4.2	4.3	5.8	5.2	<0.001
Anemia	12.9	16.6	15.1	13.1	<0.001
Minimally invasive approach (%)	40.4	37.1	42.2	44.9	<0.001
Type of resection (%)					<0.001
Right colectomy	53.5	55.1	46.7	44.4	
Transverse colectomy	5.8	5.8	5.3	4.8	
Left colectomy	9.2	12.6	10.7	11.1	
Sigmoid colectomy	19.8	16.9	23.7	26.3	
Total colectomy	2.4	2.7	2.6	2.4	
Rectal resection	9.3	6.8	11.0	11.0	
Hospital operative volume (%)					<0.001
Low volume	33.4	33.5	35.5	34.4	
Medium volume	33.1	32.7	39.8	34.5	
High volume	33.4	33.8	24.6	31.1	
Hospital region (%)					<0.001
Northeast	19.7	16.5	13.2	20.9	
Midwest	23.9	17.1	5.9	9.9	
South	38.6	58.0	42.0	27.1	
West	17.8	8.4	38.9	42.1	
Hospital teaching status (%)					<0.001
Non-metropolitan	10.9	6.3	2.8	4.3	
Metropolitan non-teaching	28.6	21.3	28.6	26.6	
Metropolitan teaching	60.5	72.4	68.6	69.1	

Overall, 488,854 (67.6 %) had *Elective* and 233,882 (32.4 %) *Emergent* admission. Over the 10-year study period, there was a modest decrease in *Emergent* operations from 33.7 % in 2011 to 32.8 % in 2020 (*p* < 0.001). Compared to *Elective* patients, *Emergent* patients were older (71 vs 67 years) and more commonly Black (13.6 vs 10.1 %) and Hispanic (9.3 vs 7.2 %, Table 2). *Emergent* patients were less frequently privately insured (22.6 vs 34.8 %) and had a higher burden of comorbidities (4.3 vs 3.4). Relative to *Elective*, *Emergent* operations were less commonly minimally invasive (24.6 vs 48.1 %), less frequent at high-volume (25.4 vs 36.1 %) and metropolitan teaching hospitals (58.3 vs 65.2 %), and more common in the South (42.3 vs 39.3 %).

**Table 2:** Patient, operative and hospital characteristics stratified by type of admission for colorectal cancer resection. IQR: interquartile range. SD: standard deviation.

Parameter	Elective (*n* = 488,854)	Emergent (*n* = 233,882)	*p*-Value
Age (years, median, IQR)	67 [57–77]	71 [60–81]	<0.001
Female sex (%)	49.0	50.7	<0.001
Race (%)			<0.001
White	76.4	70.6	
Black	10.1	13.6	
Hispanic	7.2	9.3	
Other	6.2	6.4	
Payer status (%)			<0.001
Private	34.8	22.6	
Medicare	55.8	61.3	
Medicaid	5.9	9.5	
Uninsured	1.5	4.3	
Other	2.0	2.3	
Comorbidities (%)			
Elixhauser Comorbidity Index (mean ± SD)	3.4 ± 1.7	4.3 ± 1.8	<0.001
Diabetes	23.5	24.9	<0.001
Hypertension	57.8	61.0	<0.001
Obesity	15.4	12.6	<0.001
Congestive heart failure	6.6	13.9	<0.001
Chronic liver disease	4.0	5.2	<0.001
Anemia	9.8	21.3	<0.001
Minimally invasive approach (%)	48.1	24.6	<0.001
Type of resection (%)			<0.001
Right colectomy	50.4	57.1	
Transverse colectomy	5.4	6.1	
Left colectomy	9.2	11.2	
Sigmoid colectomy	20.6	19.4	
Total colectomy	2.3	2.8	
Rectal resection	12.1	3.4	
Hospital operative volume (%)			<0.001
Low volume	30.5	40.3	
Medium volume	33.4	34.3	
High volume	36.1	25.4	
Hospital region (%)			<0.001
Northeast	18.8	19.3	
Midwest	21.8	18.9	
South	39.3	42.3	
West	20.1	19.5	
Hospital teaching status (%)			<0.001
Non-metropolitan	9.2	9.5	
Metropolitan non-teaching	25.5	32.2	
Metropolitan teaching	65.2	58.3	

**Fig. 1:**
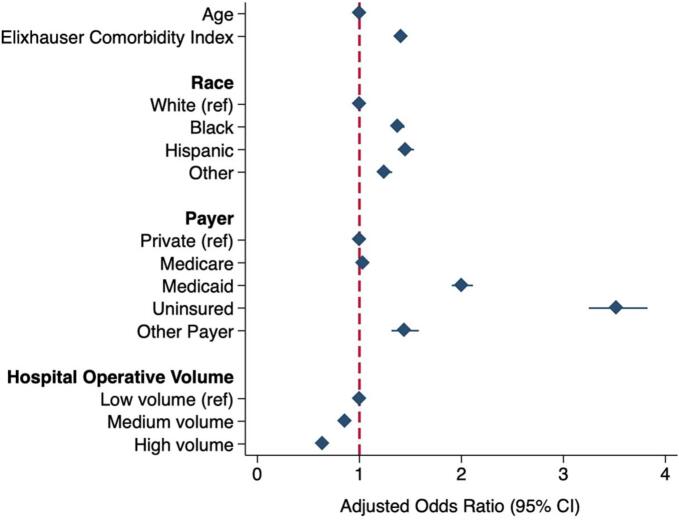
Patient and hospital characteristics associated with emergent admission for colorectal cancer resection. Model C-statistic: 0.75. Ref: reference. CI: confidence interval.

**Fig. 2:**
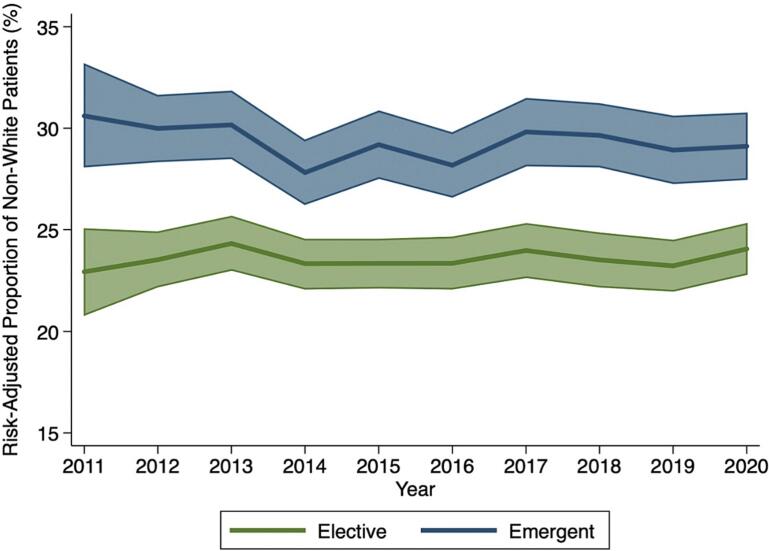
Risk-adjusted proportion of non-White patients undergoing colorectal cancer resection over the 10-year study period stratified by emergent or elective admission.

Following adjustment for the patient, operative, and hospital factors tabulated in Supplemental Table 2, Black (AOR 1.38 [95 % CI 1.33–1.44]), Hispanic (1.45 [1.38–1.53]), and Other (1.25 [1.18–1.32]) race remained associated with significantly increased odds of *Emergent* operation relative to White (Fig. 1). Trend analysis revealed that non-White patients consistently comprised over 5 % greater risk-adjusted proportion of the *Emergent* cohort compared to *Elective* over the study period (2011: 30.6 vs 22.9 %, 2020: 29.1 vs 24.1 %, Fig. 2). In addition, Medicaid (AOR 2.01 [95 % CI 1.91–2.11]) and uninsured status (3.52 [3.25–3.82]) were significantly associated with *Emergent* admission compared to private insurance. Relative to low-volume centers, medium (0.86 [0.82–0.89]) and high-volume (0.64 [0.61–0.67]) centers had significantly decreased odds of *Emergent* operation.

**Fig. 3:**
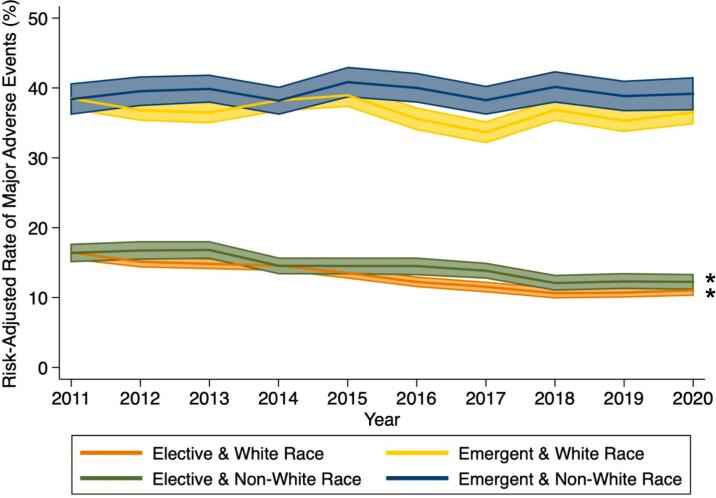
Risk-adjusted rate of major adverse events among patients undergoing colorectal cancer resection over the 10-year study period stratified by admission type and race. *p < 0.001.

Clinical and financial outcomes are shown in Table 3. Compared to *Elective, Emergent* patients had significantly greater rates of mortality (4.1 vs 0.9 %) and complications including respiratory (14.6 vs 4.4 %), infectious (14.2 vs 3.6 %), and renal (19.0 vs 5.8 %, all *p* < 0.001). The *Emergent* cohort had a significantly greater proportion of patients with Clavien-Dindo Grade IV/V severity of complications (19.0 vs 5.9 %, p < 0.001). Furthermore, LOS (11.9 vs 6.0 days), hospitalization costs ($33,900 vs 21,700), and non-home discharge (32.2 vs 10.6 %) were all significantly increased in the *Emergent* cohort (all p < 0.001). Upon risk adjustment, *Emergent* admission was associated with approximately 3-fold increase in odds of mortality, respiratory, infectious, and renal complications, Clavien-Dindo Grade IV/V complications, and non-home discharge compared to *Elective* (Table 3). Adjusted rates of MAE among *Emergent* operations remained significantly greater than *Elective* over the study period, and the gap in MAE rates widened between *Emergent* and *Elective* patients over time regardless of race (White race: 22.1 % in 2011 to 25.5 % in 2020, Non-White race: 22.0 % to 27.0 %, *p* < 0.001, Fig. 3). Of note, patients of non-White race experienced significantly increased MAE compared to White patients (Fig. 3). The disparity in MAE rates between non-White and White patients persisted since 2015 and widened in recent years regardless of admission type (Emergent: 1.9 % in 2015 to 2.7 % in 2020, Elective: 1.0 % to 1.3 %, *p* = 0.001).

**Table 3:** Clinical and financial outcomes of patients undergoing resection for colorectal cancer stratified by type of admission.

	Elective (n = 488,854)	Emergent (n = 233,882)	p-Value	AOR/ß	95 % CI
In-hospital mortality (%)	0.9	4.1	<0.001	2.92	2.68–3.19
Clavien-Dindo classification (%)			<0.001		
No complication/Grade I	25.1	10.3		0.44	0.42–0.46
Grade II	3.2	3.1		0.79	0.73–0.85
Grade III	65.8	67.6		1.08	1.05–1.12
Grade IV/V	5.9	19.0		2.67	2.57–2.78
Complications (%)					
Respiratory	4.4	14.6	<0.001	2.56	2.45–2.68
Infectious	3.6	14.2	<0.001	3.67	3.49–3.85
Renal	5.8	19.0	<0.001	2.89	2.77–3.00
Thromboembolic	0.7	3.0	<0.001	2.95	2.65–3.28
Cardiac	1.0	2.7	<0.001	1.71	1.56–1.89
LOS (days, mean, SD)	6.0 ± 5.3	11.9 ± 8.6	<0.001	4.7	4.6–4.8
Costs ($1000s, mean, SD)	21.7 ± 19.1	33.9 ± 27.7	<0.001	10.1	9.7–10.4
Non-home discharge (%)	10.6	32.2	<0.001	3.06	2.95–3.17

## Discussion

Using a nationally representative cohort of patients undergoing colorectal cancer resection, the present study examined the association of emergent admission with race as well as in-hospital clinical and financial outcomes. Relative to White race, Black, Hispanic, and other non-White races including Asian/Pacific Islander and Native American were significantly associated with increased odds of emergent operation. Over the 10-year study period, non-White patients consistently comprised over 5 % increased risk-adjusted proportion of the *Emergent* group compared to the *Elective* group. Furthermore, *Emergent* patients had significantly greater likelihood of mortality, postoperative complications, LOS, and hospitalization costs relative to *Elective*, and the disparity in MAE interestingly widened over time. Notably, non-White patients experienced persistently increased rates of MAE compared to White patients regardless of admission status. Several of these findings warrant further discussion.

Our findings add to a body of literature on racial disparities in colorectal cancer care. Prior analyses from the Surveillance, Epidemiology, and End Results database as well as the Michigan Surgical Quality Collaborative reported that Black patients, particularly those in high-poverty neighborhoods and those with public or no insurance, were more likely to receive emergent diagnosis and treatment of colorectal cancer compared to White patients [10,17]. While much of existing disparity literature focuses on White versus Black populations, we also found that Hispanic, Asian/Pacific Islander, and Native American patients experienced significantly greater odds of emergent operation in our nationwide cohort. Patients of minority race often experience lower income and have historically faced barriers in accessing regular primary care, and lack of an established primary care physician has been linked with increased likelihood of emergent colon cancer diagnosis [18,19]. In addition, we found that non-White patients more frequently presented for surgery at a younger age, signifying earlier onset of disease compared to White patients [[2], [3], [4]]. Other studies have demonstrated that non-White patients are more likely to be diagnosed at advanced stages of cancer and have incomplete preoperative oncologic evaluations [10,20]. Early recognition of colorectal cancer is crucial to timely management, and these findings suggest that targeted interventions to mitigate racial bias and address systemic racism in the access and delivery of oncologic care are warranted.

Moreover, the present study revealed that emergent admission was associated with significantly increased likelihood of adverse clinical and financial outcomes, consistent with prior literature [10,21]. As expected, non-White patients undergoing emergent operations were particularly at-risk of adverse postoperative events compared to White counterparts or those undergoing elective operations. Alleviating these racial disparities requires additional efforts to identify and address causal factors. We found that emergent colorectal resections were less commonly performed with minimally invasive approaches. Laparoscopic colectomy has previously been linked with improved mortality and complication rates compared to open colectomy [22]. Operative approach may be subject to surgeon preference and experience, availability of specialized colorectal surgeons versus general surgeons in the emergent setting, and access to appropriate surgical technology. Increased adoption of laparoscopy should be considered in the emergent setting to improve outcomes of colorectal cancer resection, particularly among racial minority patients. In addition, medium and high-volume centers had decreased odds of emergent operation in the present study, presenting another modifiable risk factor [23]. Given racial inequities in access to transportation and increased travel distance, malignant colonic perforation may still warrant immediate surgery at the nearest center [24]. However, regionalization to experienced centers may be beneficial for less urgent indications such as partial bowel obstruction.

Notably, the disparities in racial makeup and MAE between emergent and elective cohorts persisted over time. Interestingly, the disparity in MAE widened due to significant improvement among elective but not emergent patients. Nationwide adoption of Enhanced Recovery After Surgery (ERAS) protocols for elective cases in recent years has likely driven the substantial reduction in MAE. Several reports have demonstrated that implementation of ERAS programs in colorectal surgery leads to decreased morbidity and length of stay without increased readmissions [[25], [26], [27], [28], [29]]. In addition, ERAS in colorectal surgery has been shown to decrease costs and complication rates, as well as increase patient satisfaction and quality of life [[30], [31], [32], [33], [34], [35]]. Of note, use of ERAS protocols in non-elective cases is still in its infancy [36].

Another key factor likely contributing to the observed racial disparities is lack of access to screening. As routine colorectal cancer screening guidelines and technologies improve, these changes primarily benefit those with close primary care follow-up and instead exacerbate disparities for those with limited access, namely non-White patients facing emergent diagnosis and treatment [37]. Barriers to cancer screening among racial and ethnic minorities are multifactorial, including lack of health literacy and historic distrust of the healthcare system at the patient level, lack of prompt screening recommendations at the provider level, and lack of financial capital, insurance coverage, and access to primary care at the systemic level [38]. Particularly among Hispanic, Asian/Pacific Islander, and Native American populations, language barriers and lack of culturally sensitive resources often impair access to screening and cancer care [39]. Furthermore, structural racism and discrimination underlie all levels of healthcare delivery and perpetuate disparities in colorectal cancer care [40].

Given the substantial adverse outcomes associated with emergent colorectal cancer operations, addressing these racial disparities should be a key focus of quality improvement efforts. In several prior randomized trials, community-based interventions such as telephone-based outreach and patient navigators have been shown to increase rates of colorectal cancer screening and diagnosis among racial/ethnic minority and low-income populations [[41], [42], [43], [44]]. The Delaware Cancer Treatment Program was a particularly effective statewide initiative that provided coverage for screening, patient navigation for care coordination, case management, and targeted community outreach [45]. Within the first 5 years, the program eliminated disparities in colorectal cancer screening and mortality rates between Black and White patients, as well as significantly reduced the proportion of Black patients presenting with advanced disease from 79 % to 40 %. Further efforts to incorporate such interventions at the national level are warranted in order to increase access to care and improve postoperative outcomes for racial minority patients with colorectal cancer.

The present study has several limitations inherent to its retrospective nature and use of administrative data. The NIS lacks clinical granularity regarding information such as the time of cancer diagnosis, cancer staging, indication for emergent operation, and anatomic or physiologic complexity of each procedure. Outpatient data including previous colorectal cancer screening were unable to be assessed. In addition, the clinical and financial endpoints analyzed were limited to the duration of admission and long-term outcomes such as readmissions and reoperations were not available. Mortality and complications were aggregated into MAE for a composite analysis of temporal trends. However, the heterogeneity of individual complications (respiratory, infectious, renal, thromboembolic, and cardiac) with variability in clinical sequelae should be considered in interpretation of these findings. Creation of a colostomy or ileostomy was not assessed in this study and is naturally associated with its own complications, such as bowel obstruction, bleeding, parastomal hernia, and readmission from dehydration, which may all further contribute to costs associated with emergent operations [46]. Furthermore, ICD coding is often influenced by provider and center practices among participating hospitals in the NIS, and the transition from ICD-9 to ICD-10 may introduce variations in coding. Despite these limitations, we utilized the largest all-payer inpatient database and robust statistical methods to enhance the generalizability of our findings at the national level.

In conclusion, the present study used a nationally representative database to demonstrate that non-White race was significantly associated with increased odds of emergent colorectal cancer resection and major adverse events. Moreover, these racial disparities have persisted over the past decade. Our findings emphasize the need for systematic approaches to improve access to timely colorectal cancer screening and preoperative oncologic assessment to mitigate the need for emergent operation in racial minority populations.

## Funding sources

The present work did not receive any funding.

## Ethical approval statement

This study was deemed exempt from full review by the Institutional Review Board at the University of California, Los Angeles.

## CRediT authorship contribution statement

**Ayesha P. Ng:** Conceptualization, Formal analysis, Methodology, Software, Writing – original draft, Writing – review & editing. **Nam Yong Cho:** Software, Writing – review & editing. **Shineui Kim:** Conceptualization, Writing – review & editing. **Konmal Ali:** Writing – original draft. **Saad Mallick:** Writing – review & editing. **Hanjoo Lee:** Conceptualization, Supervision, Writing – review & editing. **Peyman Benharash:** Conceptualization, Project administration, Resources, Supervision, Writing – review & editing.

## Declaration of competing interest

The authors declare no conflicts of interest.

## Appendix A Supplementary data

Supplementary tables Image 1
