# Supplementary material for: National analysis of racial disparities in emergent surgery for colorectal cancer
Source: Surg Open Sci. 2024 Jan 24;18:35–41. doi: 10.1016/j.sopen.2024.01.013 (PMC10838942; doi:10.1016/j.sopen.2024.01.013)
Supplement: Supplementary file 1 — Supplementary tables [file mmc1.docx]

Supplemental Table 1. Administrative *International Classification of Diseases, 9^th^ and 10^th^ Revision* (ICD-9/10) diagnosis and procedure codes for colorectal cancer resection.

|  | **ICD-9** | **ICD-10** |
| --- | --- | --- |
| **Colectomy** |  |  |
| Right colectomy |  |  |
| Open | 45.73 | 0DBF0ZZ, 0DBK0ZZ, 0DTF0ZZ, 0DTK0ZZ |
| Laparoscopic | 17.33 | 0DBF4ZZ, 0DBK4ZZ, 0DTF4ZZ, 0DTK4ZZ |
| Left colectomy |  |  |
| Open | 45.75 | 0DBG0ZZ, 0DBM0ZZ, 0DTG0ZZ, 0DTM0ZZ |
| Laparoscopic | 17.35 | 0DBG4ZZ, 0DBM4ZZ, 0DTG4ZZ, 0DTM4ZZ |
| Transverse colectomy |  |  |
| Open | 45.74 | 0DBL0ZZ, 0DTL0ZZ |
| Laparoscopic | 17.34 | 0DBL4ZZ, 0DTL4ZZ |
| Sigmoid colectomy |  |  |
| Open | 45.76 | 0DBN0ZZ, 0DTN0ZZ |
| Laparoscopic | 17.36 | 0DBN4ZZ, 0DTN4ZZ |
| Total colectomy |  |  |
| Open | 45.71, 45.82 | 0DBE0ZZ, 0DTE0ZZ |
| Laparoscopic | 17.31, 45.81 | 0DBE4ZZ, 0DTE4ZZ |
| **Rectal Resection** |  |  |
| Open | 48.51, 48.52, 48.62 | 0DTP0ZZ, 0DBP0ZZ |
| Laparoscopic | 48.51 | 0DTP4ZZ, 0DBP4ZZ |
| **Robot-Assisted** | 17.42, 17.44, and laparoscopic procedure code | 8E0W4CZ, 8E0W8CZ, and laparoscopic procedure code |
| **Colorectal cancer** | 153, 154 | C18, C19, C20 |
|  |  |  |
| **Comorbidities** |  |  |
| Diabetes | 250 | E10, E11, E13 |
| Hypertension | 401, 402, 403, 404, 405 | I10, I11, I12, I13, I15 |
| Obesity | E66 | 278.0 |
| Congestive heart failure | 398.91, 402.01, 402.91, 404.01, 404.03, 404.11, 404.13, 404.91, 404.93, 425.4, 425.5, 425.7, 425.8, 425.9, 428 | I43, I50, I09.9, I11.0, I13.0, I25.5, I42.0, I42.5, I42.6, I42.7, I42.8, I42.9, P29.0 |
| Chronic kidney disease | 403.01, 403.11, 403.91, 404.02, 404.03, 404.12, 404.13, 404.92, 404.93, 588.0, 585.5, 585.6, 586.5, 586.6 | Z49, N19, I12.0, N25.0, Z94.0, N18.5, N18.6, I13.11, V45.1, Z99.2, Z91.15 |
| Chronic liver disease | 070.22, 070.23, 070.32, 070.33, 070.44, 070.54, 070.6, 070.9, 456.0, 456.1, 456.2, 572.2, 572.3, 572.4, 572,8, 573.3, 573.4, 573.8, 573.9, V42.7, 570, 571 | K70, K72, K73, K74, B18, I85, K76.3, K76.4, K76.5, K76.6, K76.7, K76.8, K76.9, Z94.4, K71.1, K71.3, K71.4, K71.5, K71.7, K76.0, K76.2, I86.4, I98.2 |
| Anemia | 280, 281 | D50, D51, D52, D53 |
|  |  |  |
| **Complications** |  |  |
| Respiratory Failure | 518.81, 518.51, 518.53, 518.84 | J96.00, J96.90, J96.20, J95.821, J95.822 |
| Prolonged Mechanical Ventilation | 96.72 | 5A1955Z |
| Pneumonia | 480, 481, 482, 483, 485, 486, 997.31, 997.32 | J12, J13, J14, J15, J16, J18, J95.851, J95.89 |
| Sepsis | 038, 995.91, 995.92, 999.3, 998.51, 998.59 | A40, A41, R65.20, T814XXA, K68.11 |
| Abscess | 569.5, 790.7 | R78.81, K63.0 |
| Wound infection | 998.31, 998.32, 998.5 | T81.32XA, T81.31XA, T81.4XXA, K68.11 |
| Acute Kidney Injury | 584 | N17 |
| Deep Vein Thrombosis | 451.1, 451.2, 451.81, 451.9, 453.2, 453.40, 453.41, 453.42, 453.8, 453.9 | I82.220, I82.4, I82.6, I82.A1, I82.B1, I82.C1, I82.290, I82.890, I82.91, I80.9, I80.3 |
| Pulmonary Embolism | 415.1 | I26 |
| Cardiac Arrest | 427.5 | I46.2, I46.8, I46.9 |
| Cardiogenic Shock | 785.51 | R57.0 |
| Myocardial infarction | 410 | I21 |
|  |  |  |
|  |  |  |
|  |  |  |
|  |  |  |
|  |  |  |
|  |  |  |
|  |  |  |
|  |  |  |
|  |  |  |
|  |  |  |
|  |  |  |
|  |  |  |
|  |  |  |

Supplemental Table 2. Patient, operative, and hospital characteristics associated with emergent admission for colorectal cancer resection. Model C-statistic: 0.75. *Ref: Reference. AOR: Adjusted odds ratio. CI: Confidence interval.*

| **Parameter** | **AOR [95% CI]** | ***p*-value** |
| --- | --- | --- |
| Age | 1.01 [1.00-1.01] | <0.001 |
| Female sex (ref: male) | 0.99 [0.96-1.01] | 0.42 |
| *Race* |  |  |
| White | Ref |  |
| Black | 1.38 [1.33-1.44] | <0.001 |
| Hispanic | 1.45 [1.38-1.53] | <0.001 |
| Other | 1.25 [1.18-1.32] | <0.001 |
| *Payer Status* |  |  |
| Private | Ref |  |
| Medicare | 1.04 [1.00-1.08] | 0.04 |
| Medicaid | 2.01 [1.91-2.11] | <0.001 |
| Uninsured | 3.52 [3.25-3.82] | <0.001 |
| Other | 1.44 [1.31-1.58] | <0.001 |
| *Comorbidities* |  |  |
| Elixhauser Comorbidity Index | 1.41 [1.40-1.43] | <0.001 |
| Diabetes | 0.70 [0.67-0.72] | <0.001 |
| Hypertension | 0.64 [0.61-0.67] | <0.001 |
| Obesity | 0.59 [0.57-0.61] | <0.001 |
| Congestive heart failure | 1.15 [1.10-1.20] | <0.001 |
| Chronic liver disease | 0.83 [0.78-0.88] | <0.001 |
| Anemia | 1.50 [1.45-1.56] | <0.001 |
| Minimally invasive approach | 0.38 [0.37-0.39] | <0.001 |
| *Type of Resection* |  |  |
| Right colectomy | Ref |  |
| Transverse colectomy | 0.90 [0.85-0.94] | <0.001 |
| Left colectomy | 1.07 [1.03-1.12] | 0.001 |
| Sigmoid colectomy | 0.93 [0.90-0.97] | <0.001 |
| Total colectomy | 0.99 [0.91-1.07] | 0.74 |
| Rectal resection | 0.27 [0.25-0.29] | <0.001 |
| *Hospital Operative Volume* |  |  |
| Low volume | Ref |  |
| Medium volume | 0.86 [0.82-0.89] | <0.001 |
| High volume | 0.64 [0.61-0.67] | <0.001 |
| *Hospital Region (%)* |  |  |
| Northeast | Ref |  |
| Midwest | 0.77 [0.73-0.82] | <0.001 |
| South | 1.04 [0.99-1.09] | 0.16 |
| West | 0.88 [0.84-0.94] | <0.001 |
| *Hospital Teaching Status (%)* |  |  |
| Non-metropolitan | Ref |  |
| Metropolitan non-teaching | 1.46 [1.37-1.56] | <0.001 |
| Metropolitan teaching | 1.24 [1.17-1.33] | <0.001 |
